# Load Index Metrics for an Optimized Management of Web Services: A Systematic Evaluation

**DOI:** 10.1371/journal.pone.0068819

**Published:** 2013-07-16

**Authors:** Paulo S. L. Souza, Regina H. C. Santana, Marcos J. Santana, Ed Zaluska, Bruno S. Faical, Julio C. Estrella

**Affiliations:** 1 Computer Systems Department, University of Sao Paulo, Sao Carlos, Sao Paulo, Brazil; 2 Electronic and Computer Science, University of Southampton, Southampton, United Kingdom; University of Cape Town, South Africa

## Abstract

The lack of precision to predict service performance through load indices may lead to wrong decisions regarding the use of web services, compromising service performance and raising platform cost unnecessarily. This paper presents experimental studies to qualify the behaviour of load indices in the web service context. The experiments consider three services that generate controlled and significant server demands, four levels of workload for each service and six distinct execution scenarios. The evaluation considers three relevant perspectives: the capability for representing recent workloads, the capability for predicting near-future performance and finally stability. Eight different load indices were analysed, including the *JMX Average Time* index (proposed in this paper) specifically designed to address the limitations of the other indices. A systematic approach is applied to evaluate the different load indices, considering a multiple linear regression model based on the stepwise-AIC method. The results show that the load indices studied represent the workload to some extent; however, in contrast to expectations, most of them do not exhibit a coherent correlation with service performance and this can result in stability problems. The *JMX Average Time* index is an exception, showing a stable behaviour which is tightly-coupled to the service runtime for all executions. Load indices are used to predict the service runtime and therefore their inappropriate use can lead to decisions that will impact negatively on both service performance and execution cost.

## Introduction

Web services are widely used in many Internet applications and comprise an essential component of SOA (Service-Orientated Architecture) systems. One major advantage is their intrinsic platform-independence, which becomes possible once the necessary web services are available from a server (or multiple servers) and accessed via the Internet [1], [2]. This paper presents a systematic evaluation of well-known load indices in the context of the load balancing of web services implemented on a cluster of servers.

Most web services seek to optimize key operational requirements such as high reliability, high security and shorter service runtime by adopting techniques such as load balancing [3], [4], [5], [6]. The individual server workload is used by the server management software to distribute requests from the web to individual servers in a cluster of servers. The requirement is to select the most appropriate server which will maximize the target quality achieved by an individual web service [7], [8], [9], [10], [11].

Optimum load balancing is a problem of acknowledged difficulty, because of the performance levels in web service servers which track an extremely dynamic workload generated by the requested services. Moreover, the additional load generated by middleware systems (such as Apache Tomcat [12] and JBoss [13], [14]) also affects the overall system performance. The use of these middleware systems in server clusters introduces significant differences in the overall system operation when compared with other application domains, such as HPC (High-Performance Computing), distributed databases and static-content web servers.

A load index metric typically approximates the amount of workload in an individual server. The load index definition of Ferrari and Zhou [15] is adopted in this research, defining a load index as a non-negative variable, starting from zero (an idle resource) and increasing in value as the workload increases. Such a load index frequently represents the current workload and is often used to predict the server performance expected in the near future. Several alternative indices, such as the number of requests submitted, the size of the ready-processes queue, the size of the free memory and current service runtime have been adopted by web services for this purpose [16], [17], [18], [19]. However, in many cases the metric has been adopted without an accurate evaluation of its suitability and likely efficiency. Indeed, there appears to have been little or no prior work to establish the key advantages and disadvantages of the different metrics for this application.

The main objective of this paper is to analyse qualitative aspects of the different metrics under different service demands and workload levels. The results demonstrate that a good representation of the current workload can be achieved by some indices. The ability of the load index to predict the individual server service performance in the near future is also analysed, together with the stability of the index. These are important considerations when selecting the most appropriate server in a cluster to respond to a given service request.

The research reported in this paper is part of a larger project (named Jerrymouse), designed to deliver a new framework to distribute service requests to individual servers in an optimized, dynamic and transparent fashion [20]. The features of the load indices evaluated in this study have now been incorporated in the policies developed for the Jerrymouse framework.

The experiments reported in this paper consider three specific services, representative of distinct system demands - CPU-bound, memory-bound and database applications. A number of different scenarios are considered: firstly each of the three services above (CPU-bound, memory-bound and database) running by themselves. A further scenario considers a mixture of all three services and also another two more scenarios, again with a mixture of services but running on a platform with normal load (i.e. *not-overloaded*) and then a platform which has been deliberately *overloaded*. Each of these scenarios also considers four different levels of workload, with one, two, four and then eight concurrent clients requesting the services.

A number of different load indices are analysed - seven metrics that are commonly used together with an eighth metric, the *JMX Average Time*, which has been especially designed to address the disadvantages of the other metrics. The details of this new index, the *JMX Average Time* metric, are set out for the first time in this paper.

The load indices are evaluated in this paper using three different approaches. Firstly, graphs are used to compare the measured service runtime and the values of the indices generated from a real system executing the scenarios. In this paper, service runtime is the response time, i.e., the amount of user time that has passed since a particular service started until it is completed. A systematic approach to evaluate the different load indices is then applied, using a multiple linear regression model based on the stepwise-AIC (Akaike Information Criteria) method for the selection of the best models representing an index or a union of indices [21], [22]. The service runtime was also evaluated by means of the main linear models generated through the stepwise-AIC method. This runtime prediction demonstrates, in this practical example, the impact of different load indices on the performance estimate. The final evaluation presented in this paper compares the stability of the indices based on their standard deviation. The stability of an index is compared to the other indices and, in particular, to the runtime stability.

The results show that a careful selection of metrics is essential to achieve high performance and high overall server utilization. While most of the load indices studied do indeed represent the workload to some extent, the use of a less-appropriate metric will result in the selection of an inappropriate server by incorrectly assessing a node as either overloaded or idle, when in fact a significantly more suitable selection is possible. This incorrect selection impacts both on the overall quality of service and also the energy efficiency of the system (this is the so-called “green” factor).

This paper is organized as follows: ‘SOA and Web Services’ presents concepts of SOA and web services; ‘Monitoring Web Services’ discusses how to monitor web services using the Ganglia monitoring-software tool; the main properties related to load indices are highlighted in ‘Load Indices’; ‘Materials and Method’ describes the approach used in the experiments to collect and analyse the data; ‘Results and Discussion’ points out the details of the main results, correlating their different aspects; finally, ‘Conclusions’ presents the final considerations and highlights future research directions.

### SOA and Web Services

Web services are a key mechanism for the provision of remote services executing elsewhere on the web. They allow integration between computers, databases and networks by creating a logical link that can be invoked by a distant client. Services run remotely in servers, providing functionality that is both more accessible and also independent of the platform.

Services are made available to applications through standard publishing and discovery protocols. Web service providers publish descriptions of their services using discovery agents and well-defined standards [17]. Client applications interested in these services follow the description provided to establish a connection. The WSDL (Web Service Description Language) allows the description of services by means of an XML (eXtensible Markup Language) file, which contains information about the methods supported by the service, its location and arguments. This file is stored in a broker using (for example) a UDDI (Universal Description Discovery and Integration) protocol, which defines methods to discover and publish service directories [17]. SOAP (Simple Object Access Protocol) is a protocol specification for exchanging structured information on a distributed platform, using XML content in HTTP messages. REST (Representative State Transfer) is an architectural style to build distributed applications using HTTP messages. Both REST and SOAP can be used by client applications to request remote services [23].

Web service providers are able to run in clusters, which can potentially improve a number of different aspects, such as performance, fault tolerance and availability [24], [25], [26], [27]. Apache Tomcat [12], JBoss [13], [14] and Apache Axis2 [28] are examples of web service providers. Apache Tomcat is a servlet-container fully compliant with the Oracle specification. Tomcat supports the use of clusters through automatic publication and section-replication of services which provides fault tolerance and flexibility to the client when accessing databases through dynamic pages. The automatic publication of services in different nodes assists the developer during the service publication. JBoss is designed to provide a complete solution to business-integration using Apache Tomcat and Java technology [13], [14]. Axis (Apache eXtensible Interaction System) is a framework to create clients, servers and gateways for SOAP or REST. Axis also includes a simple server to receive requests, connection with Apache Tomcat via servlets, support for WSDL updated, tools to generate classes based on WSDL and additional tools for monitoring [28].

SOA and web services have generated several new challenges in the provision of effective computing systems. These challenges can be classified into three levels: basic, composite and management [29]. The basic level refers to the details of middleware, such as publication, discovery, selection, heterogeneous platforms and security. The composite level aggregates multiple services into a single “composite” service. Orchestration and choreography are examples of interaction protocols designed to control and coordinate collaborating services. On the top of these levels is the management level, designed to provide facilities, such as service governance, monitoring, metrics and load balancing.

It is possible to organize web services in several different ways [13], [14], depending on the objectives and the choice of providers and platforms. This paper assumes that a typical platform consists of nodes acting as UDDI broker, client, front-end, back-ends and database servers. The client node hosts the application that requests services. This application searches for the service description in a UDDI broker and then sends an HTTP message with SOAP content to the front-end. The front-end can receive the HTTP message using a web server, as Apache [20] or directly through JBoss [30]. If an Apache server receives this message, it routes it to JBoss using a distributing policy to distribute requests to the back-ends. Several front-end nodes can interact and cooperate to receive requests from applications. A back-end node receives the request by using its JBoss instance, which is responsible for the actual running of the service. The back-end node returns the results to the front-end, which then sends them back to the client (back-ends may also return results directly to the client, depending on the settings). Remote database servers are usual in the web service context, where services send requests to a database in order to complete their jobs.

### Monitoring Web Services

The monitoring of web services provides useful data to estimate the near-future performance, avoid potential bottlenecks, optimize the use of system resources and generally improve the overall cost-benefit ratio [31].

Ganglia is a software-monitoring tool developed by the University of Berkeley and widely used in distributed platforms [32]. It offers scalable monitoring based on hierarchical distributions of clusters known as *federations*. Ganglia has a flexible design and can collect pre-defined indices as well as allowing users to create their own indices. The standard indices built in Ganglia provide information about the node configuration, the percentage of CPU use, the number of processes in the ready (or blocked) queue, the free memory and the use of disk and network.

Gmond is a Ganglia daemon present in all nodes. It collects and publishes previously selected indices, receives information from other nodes (using multicasting) and publishes its own information for other nodes. The information is distributed in an XML document to allow other systems in the platform to access it in a straightforward fashion, using a TCP connection and an XML parser. Gmtad is a tool that groups different gmonds, creating a hierarchical structure called *federation of clusters* (the objective of the federation concept is to reduce the overall communication overhead when updating indices over a widely-distributed platform).

The gmetric tool supports the addition of new metrics into Ganglia by means of TCP/IP messages sent to gmond. It is also able to run programs developed in other languages to collect data directly from the machine where the JSP (Java Service Provider) is running through the use of JMX (Java Management Extensions). JMX manages resources (called Managed Beans or MBeans) as applications, devices and services. Some examples of data that can be obtained from JMX are the heap size, the number of loaded classes and the number of active threads. MBeans usually runs on servers with Apache Tomcat and JBoss to monitor their nodes.

The combination of JMX and Ganglia generates and publishes load indices, providing a flexible and efficient method to monitor web service servers. JMX monitors and provides JBoss information while Ganglia is responsible for the collection and publishing of metrics. A script sends the indices collected from JBoss to gmetric, which acts as a link between these two environments: JBoss/JMX and gmetric/gmond. This arrangement enables each gmetric to send indices related to the execution of web services to gmond, which broadcasts its values to the other gmonds so that the entire cluster maintains updated information about the whole platform.

The combination of Ganglia and JMX provides a very robust framework for web services, but an important question about these monitoring tools is to determine which load index to monitor in a given specific execution. This question does not have a straightforward answer because of the diversity of demands imposed by web services. In practice, different implementations have selected different load indices to represent the web service workload. The next section describes the main load indices currently used by typical web services.

### Load Indices

The correct use of load indices must consider the overall platform objectives as well as the performance metrics associated with each objective. Some examples of overall objectives widely used are a reduction in the response time, an increase in the platform throughput, an improvement of load balancing and provision of enhanced fault tolerance [33]. Load indices collect data from a platform to quantify the overall system behaviour in the context of the objectives. They can also estimate ‘near-future’ performance based on current performance and the recent past. Service runtime reduction is a common and important objective and has been selected as the key performance objective in the experiments described in this paper to evaluate the effectiveness of the load indices.

The quality of a load index can be determined by specified criteria and desirable properties [15]: 1) sufficient accuracy to represent the workload adequately, even under different computational demands; 2) a straightforward relationship with performance metrics (ideally linear) such that the load index can be easily applied to predict the probable future performance; 3) stability, in particular by smoothing out workload peaks; 4) scalability, allowing a low implementation cost to be maintained even in large platforms. The costs of gathering the load index and the strategy adopted to transmit it to other nodes are also very important because they directly affect the scalability. This paper does not address the scalability of load indices specifically, although the Results section contains some brief comments.

Besides quantitative properties, load indices should also present other qualitative properties, such as the ability to encapsulate details from architectures and Operating Systems, a capability for supporting portability among heterogeneous platforms, and an overall objective of providing transparency to applications, providers and services. It is far from trivial to satisfy all these properties simultaneously, especially because some of them are contradictory. For example, increasing the index accuracy typically increases the cost of obtaining the data and reducing the peaks in an index usually decreases both its accuracy and ability to predict future performance.

The HPC application domain traditionally uses a number of different load indices, of which many are related to the scheduling of processes and load balancing. Some classic examples are the *number of ready processes*, *percentage of idle CPU time*, *percentage of memory used* and *number of active network connections* [34], [35], [18]. The index called *ready-processes* has been widely used in the HPC context, because it can be easily extracted from the operating system and provides important information on the overall system performance [15].

Web services typically make use of load indices to predict the performance in at least two cases: during the discovery phase and when distributing requests to the back-end nodes [36]. The discovery-phase algorithms use load indices to select the best options of services registered in brokers by providers through the UDDI protocol [24], [25], [37], [26], [30], [38], [39]. Load indices are also used after the discovery phase, when the web service provider must distribute the request that has just arrived to a back-end node in a cluster of servers [34], [16], [31], [26], [30].

A number of different load indices can be found in the scientific literature for web services [9], [40], [5]. Some examples are the number of requests (received, finalized or waiting to be attended), the shortest response time, the throughput availability (an uptime percentage for a service in a period), reliability (the probability of receiving a correct answer in a maximum specified time), safety (which represents the confidentiality and authentication guaranteed), accuracy (the service error rate) and integrity (the capability for completing the correct execution of a service transaction).

In addition to the various load indices described in the literature, service providers (e.g. JBoss [13], [14] and Tomcat [12]) often use practical indices, such as the number of submitted requests, active sessions, network traffic (among web service providers) and pending requests.

## Materials and Methods

In this experimental study services were considered on a platform consisting of three nodes with Intel Core 2 Quad 2.66 GHZ processors, 6 MB cache, 4 GB DDR2 RAM and a Sata-2 Hard Disk of 500GB/7200RPM. The database used was MySQL, version 5.1. The network was a Gigabit Ethernet with TP-LINK TL-SL3428 switch and the operating system was GNU/Linux, kernel 2.6.31–22. The compilers used were gcc 4.3 and Java 1.6.0_20. The web service provider was JBoss 5.1.0.GA. Ganglia 3.1.2 was used for the monitoring and JMX 5.1.0.GA ran on the server in default configuration.

Although relatively modest, the platform can nevertheless analyse the comparative behaviour of load indices in a web service server. It supports a controlled demand generated from the execution of applications that request services hosted in monitored servers. The application sends requests to the front-end node through HTTP messages with SOAP encapsulation. The front-end forwards the HTTP message to its local JBoss, which runs the requested service. The third node hosts a database server to handle requests from services.

The experimental study considers the execution of two different services in the front-end, generating controlled and specific workloads on the CPU and memory. A third service (called database) generates workload mainly on the memory, disks and network-interface of the remote database server to simulate the typical environment when remote databases are accessed by services [41].

These three services (CPU-bound, memory-bound and database) are used in the experimental studies to produce controlled demands to analyse the behaviour of different load indices. These controlled demands enable a deeper analysis of the behaviour of the load indices, allowing, among other things, comparing an index with respect to different demands. The demands related to CPU, memory and databases were chosen because they can be clearly correlated to real services, which generate similar workloads on the servers.

The CPU-bound service executes instructions providing arithmetic operations on integer and floating-point matrices, conditional-control structures, repetition structures and also short procedure calls [42], [43]. This service avoids I/O operation requests and the matrices used are small in order to fit into the cache and therefore avoid unnecessary main memory accesses. The CPU-bound service returns the amount of interactions and the runtime.

The memory-bound service evaluates the memory bandwidth between CPU and main memory, using vector processing with vectors large enough to eliminate cache effects and allow the description of the results in terms of a continuous bandwidth [42], [43]. The main results returned by this service are throughput, average time, shortest time and longest time.

The service designed to request the database (database service for short) is based on the OSDB benchmark, an open source version of the AS^3^AP benchmark originally developed by the Compaq Computer Corporation [41]. This service includes the creation and access of tables with data from text files and also the creation of indices.

The evaluation of the load index behaviour was performed under three different perspectives [15]: its accuracy in representing the workload, its relation with performance metrics (runtime in this case) and its stability.

The experiments do not consider a discovery phase, since this phase does not affect the load index behaviour in the front-end node. Each client-application runs exactly 1,000 iterations, each one executing the following sequence: request the service, wait until the result is returned and print the result. Each service takes approximately 10 s when running without competition from other services. The amount of 1,000 iterations for each client requesting a service running at 10 s has been chosen to avoid the inaccuracy of small samples, without generating excessive runtimes. The analyses considered confidence intervals of 99% for all the results.

One, two, four and eight concurrent application threads were requested for each service, each one in a different experiment. When four application threads are considered, for example, four concurrent requests were sent to the same service in the server. This strategy is designed to facilitate the analysis of the load indices under specific and homogeneous workloads, reducing the effect of any transitional period and allowing the real influence of each load to be determined.

Table 1 shows the load indices considered in the experiments. The indices *Idle CPU*, *Waiting I/O*, *Free Memory*, *Swap Used*, *Bytes In/Out* and *Ready Processes* were gathered directly from the operating system using the Ganglia monitoring tool. The *Amount of Requests* index represents the amount of requests recently submitted, evaluated from the difference between the last two samplings of the total amount of requests submitted, which is monitored from JMX and distributed through Ganglia.

**Table 1:** Load indices analysed in the experiments.

Indices	Descriptions
Idle CPU	Percentage of idle CPU time.
Waiting I/O	Percentage of CPU I/O waiting time.
Free Memory	Amount of GBytes free in the memory.
Swap Used	Amount of GBytes used for swap.
Bytes In/Out	Amount of KBytes received and sent over the network interface.
ReadyProcesses	Average number of processes in the ready queue (over the last minute)
Amount of Requests	Amount of requests received between the last two samples of the total amount of requests submitted, monitored by JMX.
JMX Average Time	Arithmetic average using sliding windows with the total runtime by JMX and total amount of requests submitted.
ServiceRuntime	Service runtime on the server (directly collected from the service code).

The *JMX Average Time* metric is a new load index proposed in this paper and represents the average runtime of a specific service, from a JMX perspective. As shown in Eq. (1), this index is based on the average of the five most recently-collected samples, using two sliding windows with five positions each. In Eq. (1) ***N*** is the amount of total samples already performed, ***i*** is the ***i^th^*** sample, ***n*** is the ***n^th^*** value calculated for the *JMX Average Time*, **t** is the *Service Total Runtime* by JMX and ***r*** is the *Total Amount of Requests Submitted* by JMX. The *service total runtime* by JMX is an accumulated index, containing the sum of all service runtimes since the server started operation. The *total amount of requests* represents the accumulated number of requests sent to a specific service hosted in the server since the operation started.
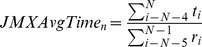
(1)


**Figure 1:**
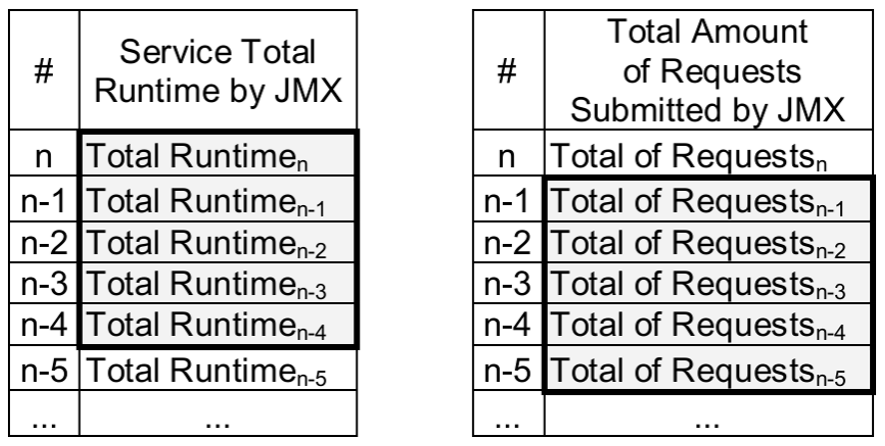
Sliding windows used to evaluate JMX Average Time.

The experimental studies performed to determine the sliding window size considered the three services (CPU-bound, Memory-Bound and Database) and sizes of windows varying from one to twenty-five positions. The standard deviation from *JMX Average Time* results for each window size was used to compare their stability. The results show that sliding windows between two and twenty-five positions were, on average, 18.9% more stable that the sliding window with just one-position, and that from five-positions it was already possible to obtain an stability 18% better. The choice of a five-position window considered the trade-off between reducing performance peaks and maintaining recent data for the calculation of *JMX Average Time*. The *JMX Average Time* index is calculated assuming a shift of one location between the samples collected for the *amount of requests* and *service total runtime* metrics, as shown in Figure 1. The *total amount of requests* metric does not consider the last sample because it represents only the requests submitted and not those already completed while the *service total runtime* represents the runtime of finalized requests. This shift minimizes the impact of those requests submitted and not yet completed. Although this mechanism does not guarantee that the average will reflect only completed requests, nevertheless it acts to minimize this problem. The empirical results demonstrate that the *JMX Average Time* metric provides an excellent index when compared with the service runtime gathered directly from the server.

Service Runtime is the response variable used in the linear models (described in next section) and was used to benchmark the behaviour of the indices being studied, because the key performance objective is to reduce the overall service runtime.

Different numbers of instances from these benchmarks were considered to represent the number of users in the system at a given moment (one, two, four and eight users). While these benchmarks were requesting services for the server, the Ganglia distributed monitoring system [32] and the Java Management Extensions (JMX) [44] kept collecting distinct load indices from the server nodes. The daemon gmond, running in the front-end node, sampled the indices every 10 s and then sent the results back to the client node. A script in the client node collected all the published indices and stored them in a log file. The interval of 10 s reduces the cost of updating the load indices and thus has minimal effect on the service runtime. In summary, the requests are sent by the clients to the servers that execute the services and are monitored by the Ganglia and JMX in order to obtain the samples.

All results of the load indices presented in this paper use the EMA (Exponential Moving Average) moving average using a window covering the last five samplings [45]. The EMA presented in Eq. (2) is similar to the simple moving average, although more weight is given to the latest data. In Eq. (2) ***V_n_*** is the last sampling performed at time ***n*** and ***N*** is the window size (five in this paper).
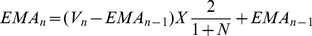
(2)


## Results and Discussion

The main results from the experiments are initially organized by service in this section. They are presented with a focus on three main factors: the relationship with service runtime (the performance objective adopted), stability and capability to represent the actual workload.

The relationship between the runtime service and the eight load indices described in Table 2 was evaluated through multiple linear regression models, which helps to understand how these indices may explain the service runtimes. In models of multiple regression it is necessary to select which predictor variables (the load indices in this paper) best explain the response variable (the service runtime). In other words, the objective is to select and rank the load indices depending on how well they explain the runtime variations. The combination of load indices that best represents the service runtime was selected using the stepwise method with AIC (the Akaike Information Criteria) [21], [22]. The stepwise method uses an automatic approach to select predictor variables, instead of considering all possible regressions. It starts with no input predictor variable in the model and in each step a new variable is introduced and then tested to see if a better model has been obtained. When the model reaches three or more predictor variables, the stepwise method checks to see if a better result can be obtained by removing one of them (the AIC criterion is used to compare the quality of the models in each step). It is important to observe that the stepwise method is able to analyse both an isolated index and merged ones during its analysis. This procedure is effectively constructing a new metric that considers an average value using distinct indices.

**Table 2:** Significance of the indices to explain the variability in the service runtimes evaluated through the stepwise-AIC method.

Load Indices	CPU-boundEstimates (%)	Memory-boundEstimates (%)	DatabaseEstimates (%)	All ServicesEstimates (%)	*Overloaded* PlatformEstimates (%)	*Not-Overloaded*Platform Estimates (%)
Idle CPU	−0.218 (3.3)	−2.843 (5.4)	−0.386 (13.6)	−0.161 (0.3)	−3.495 (6.1)	−1.579 (7.1)
CPU Waiting I/O	0.248 (3.8)	2.255 (4.3)	0.181 (6.3)	3.128 (6.0)	1.949 (3.4)	0.314 (1.4)
Swap Used	0.0 (0.0)	7.929 (15.0)	0.0 (0.0)	4.558 (8.7)	16.236 (28.2)	0.0 (0.0)
Free Memory	−0.510 (7.7)	−2.334 (4.4)	−0.074 (2.6)	−0.900 (1.7)	−0.579 (1.0)	−0.886 (4.0)
Ready Processes	0.392 (6.0)	2.399 (4.5)	0.025 (0.9)	1.836 (3.5)	0.933 (1.6)	1.421 (6.4)
Bytes In/Out	0.065 (1.0)	0.841 (1.6)	0.650 (22.8)	0.0 (0.0)	5.778 (10.1)	0.230 (1.0)
JMX Average Time	5.153 (78.2)	33.773 (64.0)	1.378 (48.3)	40.665 (77.6)	26.425 (46.0)	16.375 (74.1)
Amount of Requests	0.0 (0.0)	0.383 (0.7)	0.157 (5.5)	1.132 (2.2)	2.080 (3.6)	1.285 (5.8)

Load indices are expressed using different scales, therefore to enable a valid comparison of different indices in a common range, they were all normalized to be between -1 (lowest) and 1 (highest). The results presented in this paper for linear regression models were obtained using the *Action* statistical software package [46].

The stepwise method with AIC applied to the load indices estimates the best models to represent the service runtime. Table 2 shows the results of the influence estimated for each index, quantifying how significant the indices are (in relation to other ones) to explain the variability of the service runtimes. In this case, the higher the absolute value the more significant an index is. A positive sign indicates a direct correlation between the index and the service runtime; a negative sign indicates an inverse correlation. The load indices with estimates 0.0 (zero) in Table 2 mean that they have been discarded by the linear regression model, as they have not contributed to the service runtime prediction.

**Figure 2:**
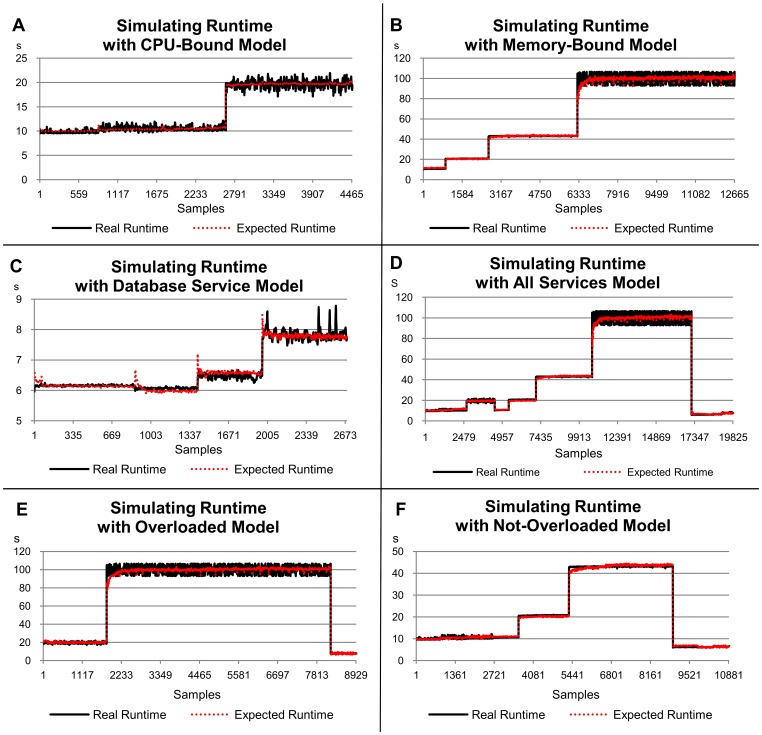
Graphs showing the relation between the predicted runtimes based on multiple linear models (used in the six scenarios) and the actual runtimes. The x-axis represents the samples collected every 10 s by Ganglia and JMX while the benchmarks were requesting services for the server.

The coefficients of determination (R^2^) shown in Table 3 quantify the amount of the variability, i.e. the extent that the runtime services (y-axis) can be predicted by the load indices (x-axis). R^2^ ranges from 0 to 1, where values close to 1 represent the best predictions. Figure 2 complements Table 3 showing graphically how the predicted service runtimes fit to the real service runtimes, using the multiple linear regression model evaluated for each one of the six scenarios.

**Table 3:** Coefficient of determination (R^2^) obtained from the stepwise-AIC method.

Scenarios	R^2^
CPU-bound	0.986
Memory-bound	0.993
Database	0.963
All services	0.996
All services with *overloaded* platform	0.991
All services with *not-overloaded* platform	0.998

The experiments described in this paper were based on six distinct execution scenarios. Firstly, the selection of models was applied separately to the results of each service: CPU-bound, memory-bound and database (the first, second and third scenarios). The objective was to identify significant differences in the behaviour of load indices when the demand requested by the service is previously known. The results from all services were then grouped and a new selection of the linear model was conducted, aiming at verifying the behaviour of the indices when requests are performed for services whose demand is heterogeneous and normally unknown in advance (the fourth scenario). In this case, the evaluation considered all the results of the services with one, two, four and eight concurrent clients. A final evaluation was conducted with all three services executing in both *overloaded* and *not-overloaded* platforms (the fifth and sixth scenarios). The *overloaded* platform was based on the results from eight concurrent clients requesting the three services. In contrast, the *not-overloaded* platform used results from one, two and four concurrent clients. The term *not-overloaded* is used instead of *idle* because the platform is actually executing services requested by four concurrent clients, although it is operating normally and is not yet overloaded. During our experiments, the *overloaded* platform had on average: 73% of CPU usage, 600 MB of swap utilization, 5.3 requests being processed and 6.5 ready processes waiting in the queue. In contrast, the *not-overloaded* platform had around 48% of CPU usage, 2.2 requests being processed, 2.9 ready processes waiting in the queue and no swap space was required.

The stability of the indices was analysed with their standard deviations, using the normalized values as a basis (Table 4). These normalized values were used to permit the comparison of the standard deviations obtained for each load index and also for the service runtime. The standard deviation for a specific service represents the arithmetic average of the standard deviations from each group of clients (one, two, four and eight). Analogously, the standard deviation for all services is the arithmetic average of their standard deviation; for an *overloaded* platform it is the average of standard deviation from the results from all services with only eight concurrent clients and for the *not-overloaded* platform it is the average of standard deviation from all services with one, two and four clients.

**Table 4:** Standard deviation (SD) representing the stability of the load indices.

Scenarios	Clients	ServiceRuntime	IdleCPU	CPUWaiting I/O	SwapUsed	FreeMemory	ReadyProcesses	BytesIn/Out	JMX AverageTime	Amount ofRequests
CPU-bound	1	0.005	0.012	0.000	0.000	0.009	0.042	0.005	0.001	0.021
	2	0.009	0.059	0.001	0.000	0.011	0.058	0.017	0.001	0.041
	4	0.007	0.128	0.001	0.000	0.014	0.131	0.027	0.002	0.079
	8	0.014	0.075	0.003	0.000	0.029	0.141	0.103	0.002	0.140
	Average SD	0.009	0.069	0.001	0.000	0.016	0.093	0.038	0.001	0.070
Memory-bound	1	0.001	0.015	0.001	0.000	0.023	0.047	0.009	0.001	0.020
	2	0.002	0.016	0.001	0.000	0.043	0.061	0.009	0.004	0.035
	4	0.002	0.029	0.004	0.000	0.091	0.099	0.023	0.024	0.065
	8	0.071	0.157	0.329	0.068	0.330	0.277	0.019	0.057	0.178
	Average SD	0.019	0.054	0.084	0.068	0.122	0.121	0.015	0.021	0.074
Database	1	0.000	0.038	0.045	0.000	0.044	0.052	0.011	0.000	0.020
	2	0.001	0.024	0.000	0.000	0.013	0.061	0.006	0.002	0.053
	4	0.002	0.034	0.001	0.000	0.011	0.096	0.037	0.001	0.089
	8	0.004	0.043	0.006	0.000	0.012	0.134	0.094	0.001	0.168
	Average SD	0.002	0.035	0.013	0.000	0.020	0.086	0.037	0.001	0.083
All services		0.010	0.053	0.033	0.068	0.052	0.100	0.030	0.008	0.076
*Overloaded*		0.030	0.092	0.112	0.068	0.124	0.184	0.072	0.020	0.162
*Not- Overloaded*		0.003	0.039	0.006	0.000	0.029	0.072	0.016	0.004	0.047

The standard deviations could also be used to compose new load indices, in order to include the variability of them when estimating future runtimes. However, this would require new experimental studies with an extra dimension of complexity. Given our initial objectives and the results already achieved, this additional complexity is outside the scope of the current paper.

The analysis of the capability of load indices to represent the workload, independently of their correlation with the runtime service, was done observing the behaviour of each index when there was variation in the workload arising from changes in the number of concurrent clients.

Finally, the results are shown with the use of different (and specific) load indices to predict the service runtime. The main objective here is to demonstrate the impact of the load indices on the quality of the decisions made by the policies controlling the distribution of requests for web services. This simulation of the service runtime also verifies the coherence of the load index representation (as pointed out with the linear models). According to the multiple regression models, the more significant indices are expected to estimate the service runtimes more precisely.

The data for the indices was collected without any specific instrumentation of services, providers or applications. This approach provides for a high portability among different platforms, since these indices are the usual ones normally available in a variety of different architectures and operating systems.

Scalability and the overall costs necessary to obtain the load indices are not considered in this paper. Ganglia gathered the indices locally from the Operating System or JMX and then the normal hierarchical federation structure was used to minimize the publishing costs to remote nodes.

### CPU-Bound Service Scenario

**Figure 3:**
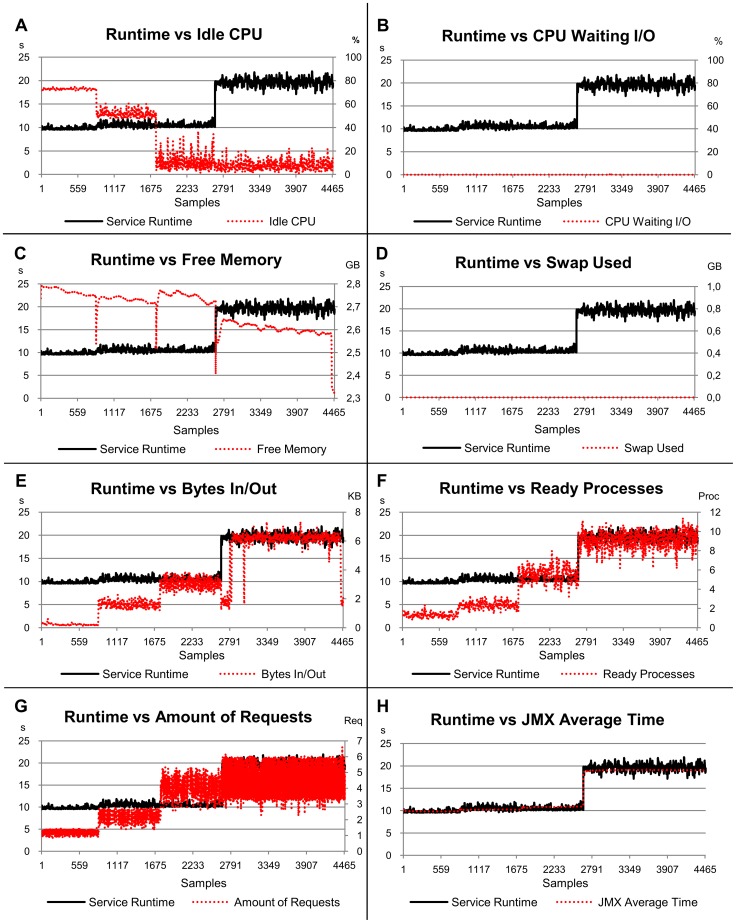
Relation of the service runtime versus eight load indices, considering the first scenario (CPU-bound service). The left-hand scale of the y-axis always represents the service runtimes in seconds (s). The right-hand scale of the y-axis, where necessary, represents the metric used by the load index. The x-axis represents the samples collected every 10 s by Ganglia and JMX while the benchmarks were requesting services for the server.

The runtimes for the CPU-bound service showed significant changes only when eight concurrent requests arrived in the server (Figure 3). The times observed scale from approximately 10 s for one, two and four clients to approximately 20 s for eight clients. This can be explained by the use of a CPU with four cores in the server hosting the service. Considering a demand located inside the CPU, each core executes a service separately up to four simultaneous requests, without any significant changes in the runtime. When there was a larger amount of services than cores (two services per core on average), the runtime doubled.

The R^2^ value for this CPU-bound service indicates that 98.6% of the runtimes can be explained by the analysed load indices (Table 3). The *JMX Average Time* load index has a strong weight in the evaluation and represents 78% of the estimates, when compared to other indices (which have a much smaller significance compared with the *JMX Average Time* metric).

This small significance is, in part, due to the behaviour of these indices when the workload changes, as a consequence of the number of concurrent clients. It is possible to observe in the graphs that the variability in the indices, excluding the *JMX Average Time*, do not follow the same behaviour as the service runtime. In contrast to expectations, even load indices strongly related to the CPU use, such as *Idle CPU* and *Ready Processes*, had a low correlation. Again, the CPU with four cores in the server was responsible for this gap, when considering CPU indices. Another aspect related to *Idle CPU* and *Ready Processes* is their low stability, according to the standard deviation given in Table 4. Similarly, the *Amount of Requests* index demonstrated high instability in this experiment and was not significant to justify most of the runtimes.

In respect of the stability, the *JMX Average Time* index also provided the best result (0.001), with an even better value than the one observed for the service runtimes. Another index with a similar result is the *CPU Waiting I/O*, mainly due to the small number of memory accesses. The standard deviation for the *Swap Used* index was zero because it was not used, hence there was no variation during executions (Table 4). The most unstable index was the *Ready Processes* index (0.093), being 10 times more unstable than the service runtime.

The load indices exhibited distinct behaviours, when analysed under the perspective of workload representation and independently of the service runtime. The *CPU Waiting I/O*, *Free Memory* and *Swap Used* indices were not able to represent the workload variations imposed by CPU-bound service. They remained at around the same level for all workloads. The *Idle CPU* index was not able to represent the change of workload from four to eight clients, since the percentage of idleness approached zero. The *Bytes In/Out*, *Ready Processes* and *Amount of Requests* indices showed variations according to the workloads submitted, although such variations did not always represent changes in the service performance in terms of runtime. As expected, the *JMX Average Time* index followed the runtime and represented the workload variation only when it affected the service performance.

### Memory-Bound Service Scenario

**Figure 4:**
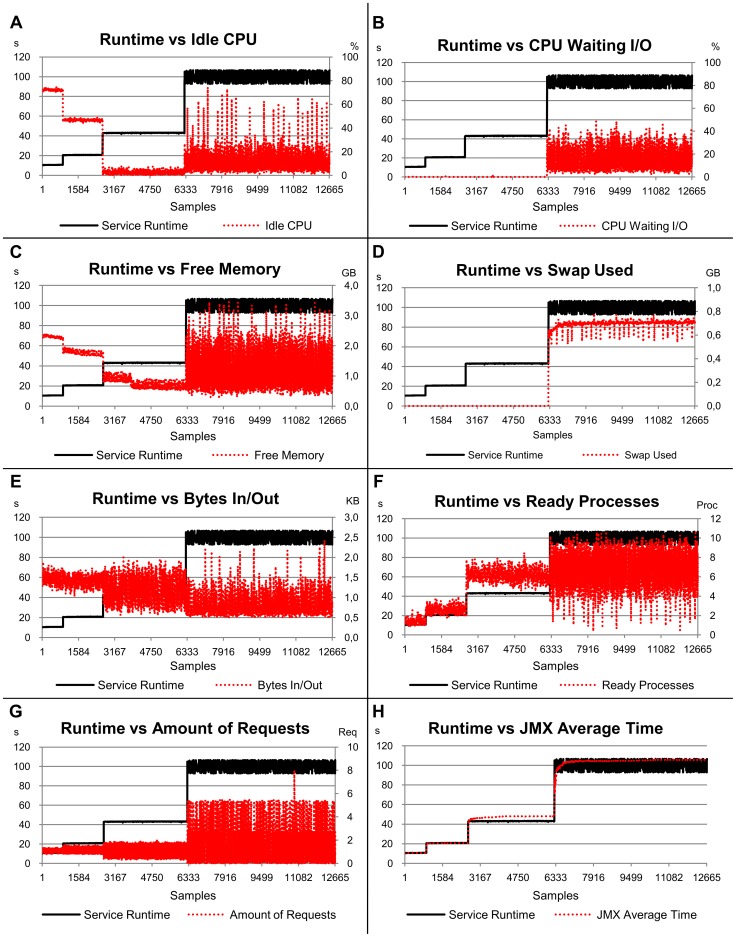
Relation of the service runtime versus eight load indices, considering the second scenario (memory-bound service). The left-hand scale of the y-axis always represents the service runtimes in seconds (s). The right-hand scale of the y-axis, where necessary, represents the metric used by the load index. The x-axis represents the samples collected every 10 s by Ganglia and JMX while the benchmarks were requesting services for the server.

Figure 4 shows that the runtimes for the memory-bound service exhibited variations for all the different amounts of concurrent clients executed. The average times were 11 s for one client, 20 s for two clients, 43 s for four clients and 100 s for eight clients. These variations occur mainly because the runtimes of the services executed in the CPU with four cores depended on the memory response, which is unique and shared by all cores. Therefore there is a gradual variation in the runtime according to the number of concurrent services in the cores.

The linear models associated with the indices analysed for the memory-bound service show that 99.3% of the runtimes can be explained by the variability in the indices (Table 3). In this context, the *JMX Average Time* index represented 64% of the estimates made and *Swap Used* 15%. These results are expected, especially the *Swap Used*, given the high memory demand generated by eight concurrent clients. However, differently from what was expected, the *Free Memory* index did not provide a good service runtime estimate, representing only 4.4%. Although the *Free Memory* index has exhibited good behaviour in respect of the computational workload, the lack of stability with four and mainly eight clients reduces its significance.

The most stable indices for the memory-bound service were *Bytes In/Out* and *JMX Average Time*, with standard deviations of 0.015 and 0.021 respectively (Table 4). These results are close to the Service Runtime stability, whose standard deviation was 0.019. A high instability of the load indices was observed, mainly when the platform remained *overloaded*. The *Swap Used* index, for example, did not show any variation with one, two and four clients; however, it presented a high instability with 8 clients (0.068). The memory-bound service with eight concurrent clients was the only case in which any memory swap was necessary in the experiments. The most unstable indices were the *Free Memory*, *Ready Processes* and the *CPU Waiting I/O* indices with standard deviation of 0.122, 0.121 and 0.084 respectively. Note that these values are up to 5.4 times higher than that observed for the service runtime (0.019). One positive aspect of this high instability is the potential ability to use it as a heuristic to detect computer overload with high-memory-demand applications.

In respect of the workload variation, the *Idle CPU*, *Free Memory*, *Ready Processes* and *JMX Average Time* indices exhibited behaviour according to the workload submitted (Figures 4–A, 4–C, 4–F and 4–H). On the other hand, the *CPU Waiting I/O*, *Swap Used* and *Amount of Requests* indices (Figures 4–B, 4–D and 4–G) remained at their normal levels with one, two and four clients, presenting a significant variability only with eight concurrent clients, when a high instability could be observed. The *Bytes In/Out* index (Figure 4–E) remained at the same level until there were four concurrent clients, however it was possible to observe a higher instability for four and eight clients.

### Database Service Scenario

**Figure 5:**
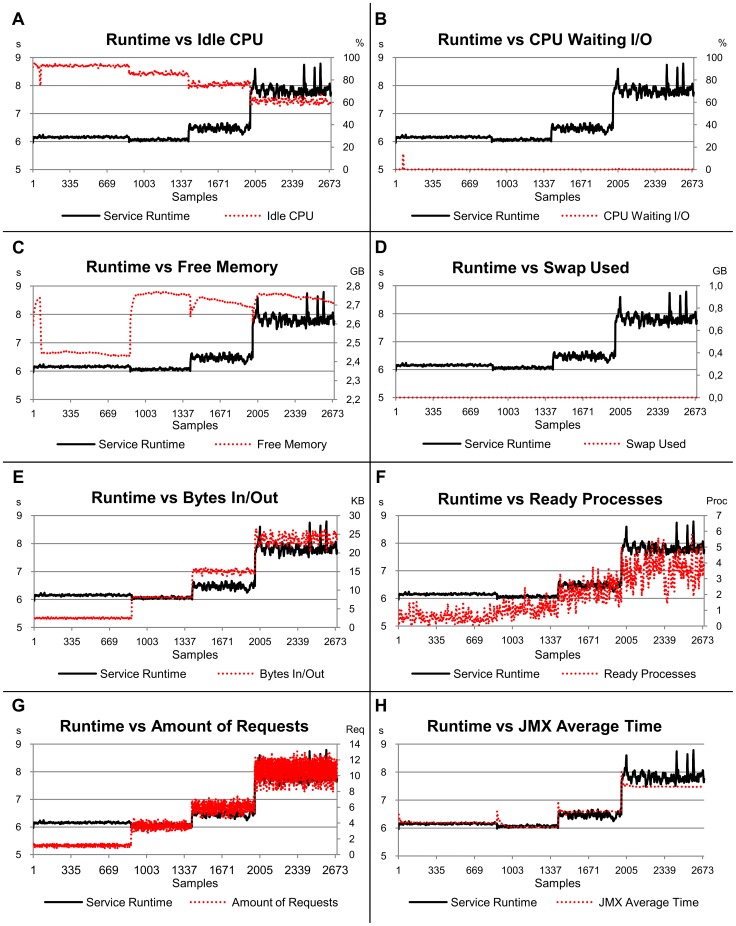
Relation of the service runtime versus eight load indices, considering the third scenario (database-bound service). The left-hand scale of the y-axis always represents the service runtimes in seconds (s). The right-hand scale of the y-axis, where necessary, represents the metric used by the load index. The x-axis represents the samples collected every 10 s by Ganglia and JMX while the benchmarks were requesting services for the server.

The service runtimes for the database service were, on average, 6.2 s, 6.1 s, 6.5 s and 7.8 s for one, two, four and eight concurrent clients respectively (Figure 5). The runtimes for one and two clients were similar mainly due to the persistent connections used by OSDB [41]. The service runtime with one and two clients is determined by the establishment of this connection and from four simultaneous accesses the demand generated was capable of affecting the service runtime.

The R^2^ for the database service indicates that 96.3% of the service runtimes are explained by the load indices analysed (Table 3). The *JMX Average Time* index had the largest representation of the estimates with 48.2%, followed by *Bytes In/Out* with 22.8%. Together these indices represent 71% of the estimates. The more significant value reached by *Bytes In/Out* for this service is coherent with the demand generated, since the database server is in a remote computer. The model also showed a representation of 13.6% for *Idle CPU* (the best result for this index) probably due to the stability observed (when comparing its stability with other services). All other indices showed a lower representation due to their behaviour in relation to the respective runtime changes (see *CPU Waiting I/O*, *Free Memory* and *Swap Used*) and their instability (see *Ready Processes* and *Amount of Requests*).

Considering only the stability (Table 4), the *JMX Average Time* index demonstrated the best standard deviation (0.001), with a value close to the service runtime (0.002). The *Swap Used* index did not show any variation during the executions with database service and therefore its value is zero. The most unstable indices were *Ready Processes* and *Amount of Requests*, with 0.086 and 0.083, respectively. These values are up to 42 times higher than that observed for the service runtime.

The *Idle CPU*, *Bytes In/Out*, *Ready Processes*, *Amount of Requests* and *JMX Average Time* indices were all able to represent the workload variation (Figures 5–A, 5–E, 5–F, 5–G and 5–H). The *CPU Waiting I/O* and *Swap Used* indices did not show any variability during the execution of the service (Figures 5–B and 5–D). The behaviour of the *Free Memory* index was not consistent with the variability in the workload (Figure 5–C).

### All Services Scenario

**Figure 6:**
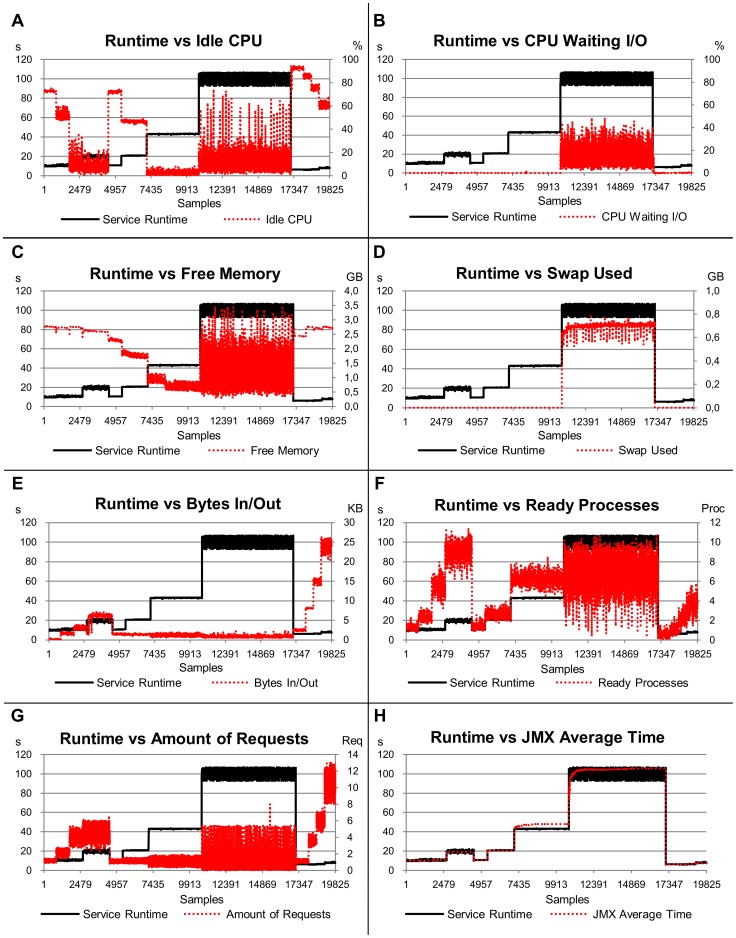
Relation of the service runtime versus eight load indices, considering the fourth scenario (all services). The left-hand scale of the y-axis always represents the service runtimes in seconds (s). The right-hand scale of the y-axis, where necessary, represents the metric used by the load index. The x-axis represents the samples collected every 10 s by Ganglia and JMX while the benchmarks were requesting services for the server.

The graphs relating runtimes of all services with the load indices are shown in Figure 6. The workload plotted in Figure 6 changes according to the service being executed (samples 1 to 4467 represent the CPU-bound service workload, samples 4468 to 17141 represent the memory-bound service workload and samples 17142 to 19825 represent the database service workload). The R^2^ resulting from the linear model with all services indicates that 99.6% of the service runtimes can be explained by the variability in the indices (Table 3). The most significant index for the estimates was again the *JMX Average Time* index, responsible for 77.6% of them. Figure 6–H shows the behaviour of this index in relation to service runtime. Despite the lower significance, the *Swap Used* index was the second best index, with 8.7%, due to its behaviour when the platform remained *overloaded*.

The most stable index for all-services was *JMX Average Time*, with a standard deviation of 0.008 (Table 4). This stability is close (and even better) to that observed for service runtime (0.010). The most unstable indices were *Ready Processes* and *Amount of Requests*, with values of 0.1 and 0.076, respectively.

### Overloaded and Not-Overloaded Platforms Scenarios

**Figure 7:**
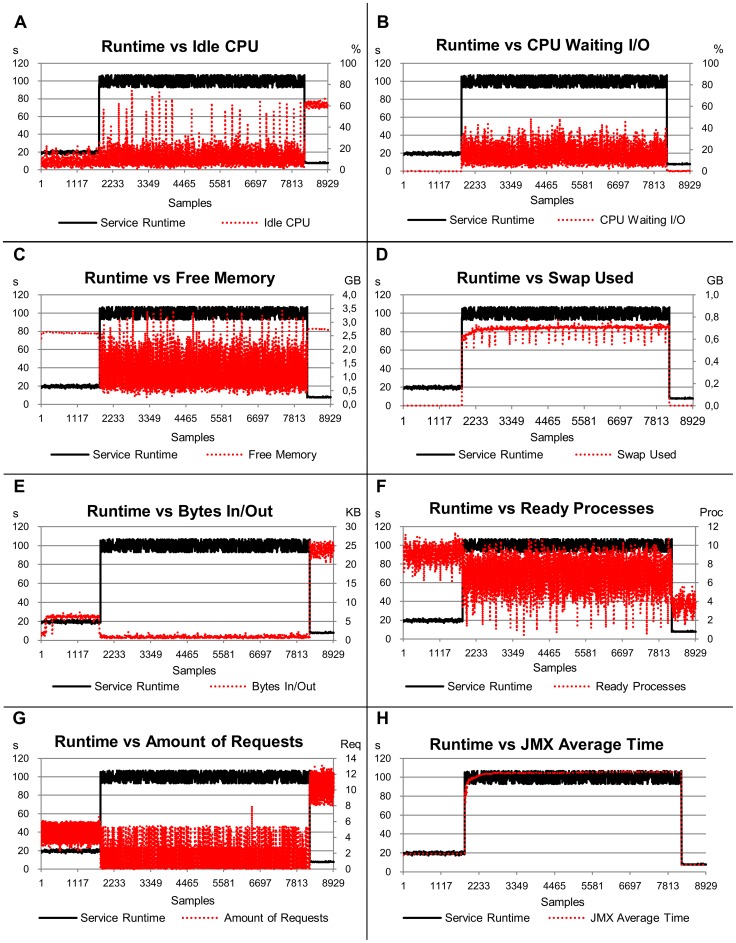
Relation of the service runtime versus eight load indices, considering the fifth scenario (overloaded platform). The left-hand scale of the y-axis always represents the service runtimes in seconds (s). The right-hand scale of the y-axis, where necessary, represents the metric used by the load index. The x-axis represents the samples collected every 10 s by Ganglia and JMX while the benchmarks were requesting services for the server.

R^2^ for the *overloaded* platform indicates that 99.1% of the variability in the indices explain the runtimes (see Table 3 and Figure 7). The *JMX Average Time* index corresponds to 46% of these estimates, followed by *Swap Used* with 28.2%. These results were as expected, due to the tight-coupling of the *JMX Average Time* index with the service runtime and the behaviour of the *Swap Used* index when executing the memory-bound service with the *overloaded* platform. It is important to point out that *Swap Used* indicated that there was no variability for the CPU-bound and database services, even with an *overloaded* platform. Another aspect verified in the experiments was the relative instability of the indices in this high-demand scenario. *Ready Processes* and *Amount of Requests* were the more unstable indices, 0.184 and 0.162 respectively (Table 4). These results are up to 8 times higher than that of the standard deviation for *JMX Average Time* (0.020). The relationships of service runtimes with *Ready Processes* and *JMX Average Time* for *overloaded* platforms are shown in Figures 7–F and 7–H.

**Figure 8:**
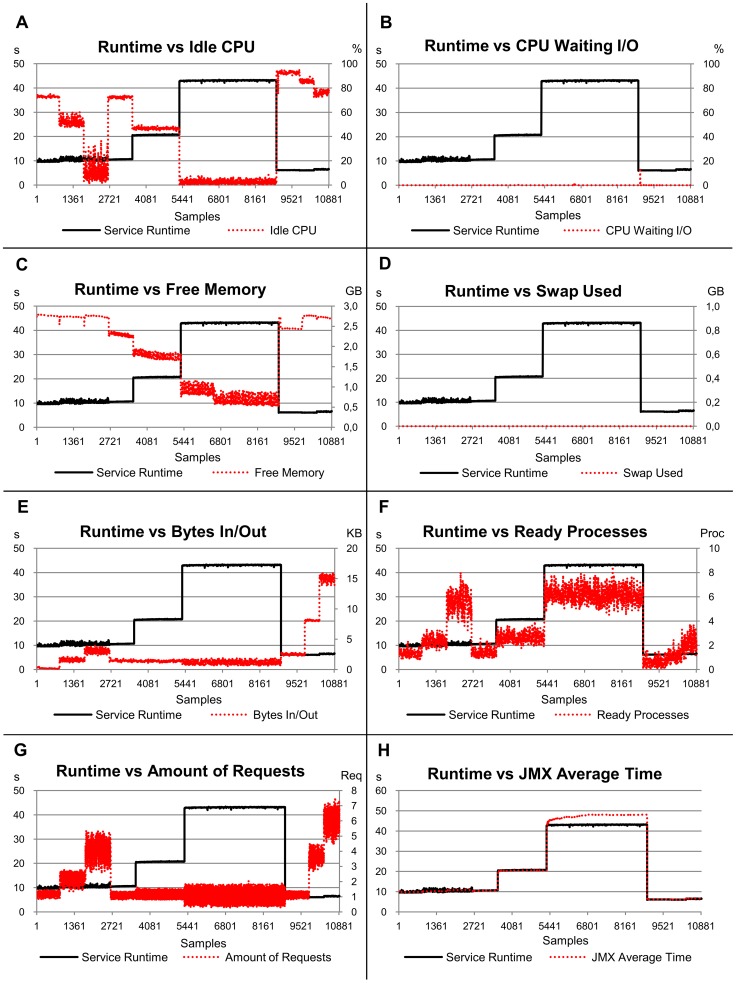
Relation of the service runtime versus eight load indices, considering the sixth scenario (not-overloaded platform). The left-hand scale of the y-axis always represents the service runtimes in seconds (s). The right-hand scale of the y-axis, where necessary, represents the metric used by the load index. The x-axis represents the samples collected every 10 s by Ganglia and JMX while the benchmarks were requesting services for the server.

The value of R^2^ for the *not-overloaded* platform was 99.8% and the *JMX Average Time* index was responsible for 74.1% of the estimates. Its significance was much larger than that of other indices in this scenario (see Figure 8 and Table 2).

In relation to stability, the *JMX Average Time* index showed a stable behaviour with a standard deviation of 0.004 (Table 4). This stability was close to that observed for the service runtime (0.003). The *CPU Waiting I/O* index also showed a stable behaviour for *not-overloaded* platforms, with a standard deviation of 0.006. However, this index presented a significance of just 1.4% to explain the variability of service runtimes. The instability of the other indices was high when executing on a *not-overloaded* platform, if compared to the variability of the service runtime. *Bytes In/Out* was 4.3 times more unstable and *Ready Processes* was 23 times more unstable than service runtime. The graphs for *JMX Average Time*, *Bytes In/Out* and *Ready Processes* (Figures 8–E, 8–F and 8–H) demonstrate the relation of these indices with service runtime.

### Predicting Service Runtimes

**Figure 9:**
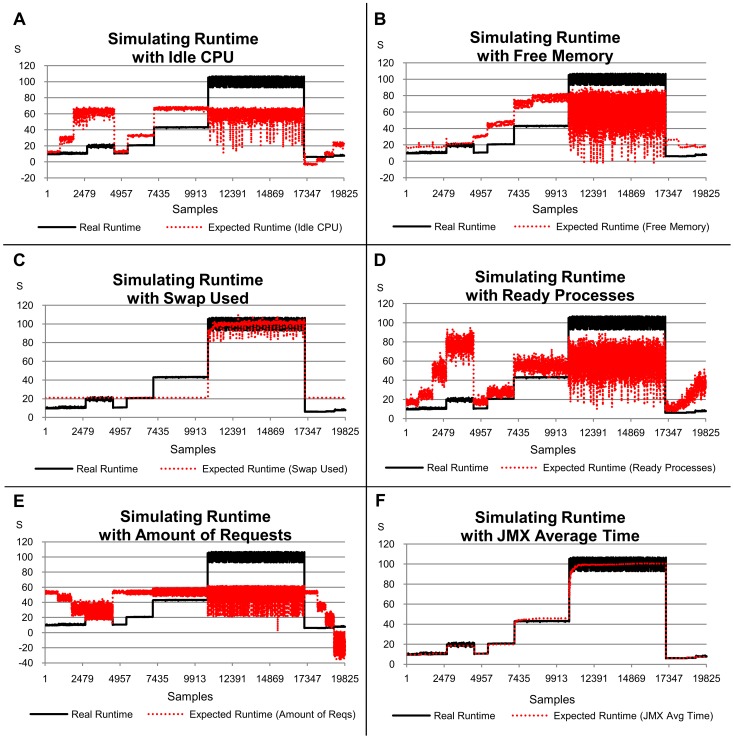
Graphs showing the relation between actual runtimes and the predicted ones. The x-axis represents the samples collected every 10 s by Ganglia and JMX while the benchmarks were requesting services for the server.

Future service runtimes were simulated in this study using simple linear models based on load indices and the correlation between these simulated times with the actual ones was analysed using the R^2^ values. The linear equations used in this simulation and the R^2^ for each of them are given in Table 5. Figure 9 shows the relation between the real service runtime (measured from the system) and the estimated service runtime (obtained from linear equations) graphically. As in this case the indices are considered individually, the normalization applied in the previous section is not necessary and the equations presented in Table 5 use the original data.

**Table 5:** Linear equations used to estimate the service runtimes and their coefficients of determination.

Load Indices	Linear Equations	R^2^
JMX Average Time	0.9473 * JMX Average Time +0.3677	0.995
Swap Used	112.9882 * Swap Used +21.0953	0.903
Idle CPU	−0.7736 * Idle CPU +68.9285	0.353
Free Memory	−29.3253 * Free Memory +97.9967	0.400
Ready Processes	7.7600 * Ready Processes +6.7807	0.301
Amount of Requests	−7.5027 * Amount of Requests +61.8613	0.201

The main objective is to demonstrate how efficient each load index would be when applied to a distributing policy to select the best server to execute a web service. Although the load indices can be used in different ways by distributing policies, this simulation shows a usable result when predicting the near-future performance, assuming a linear relation between the runtime and indices.

The service runtime simulation is based on the execution of different services generating heterogeneous demands with distinct workloads (one, two, four and eight concurrent clients). Six load indices were chosen according to their importance in relation to the estimates evaluated in the previous section. Another factor considered was the constant use of such indices as metrics by policies to distribute requests on clusters of web services.

The indices chosen were *JMX Average Time* and *Swap Used* (these were the more significant according to the linear model); *Idle CPU* and *Free Memory* (less significant according to the linear model) and *Ready Processes* and *Amount of Requests* (frequently associated with the request distributing policies).

The simulations of runtime service using *JMX Average Time* and *Swap Used* provided the best results, according to R^2^ (Table 5 and Figure 9). These results are consistent with the preliminary results from the linear regression. The simulations resulting from the *Idle CPU* and *Free Memory* indices are significantly different from the actual values observed for the service runtime, with a R^2^ of 0.353 and 0.4, respectively. The simulations resulting from *Ready Processes* and *Amount of Requests* showed results even more different from real ones, with R^2^ of 0.301 and 0.201 respectively. Divergent results mean that the policies are highly likely to make incorrect decisions, over or under estimating the host performance. These cases affect the service performance adversely and increase the overall cost of execution.

### Conclusions

This paper has described experimental studies carried out to evaluate the behaviour of eight load indices widely used for web service servers. Three perspectives were considered: 1) the capability for predicting near-future performance (using the service runtime to determine performance), 2) the workload representation and 3) index stability.

The experiments considered three different types of services that generated controlled demands on the server, four levels of workload for each service and six distinct execution scenarios, where each scenario involves: one type of service by itself, all services operating together and the level of workload in the node (*overloaded* and *not-overloaded*).

The results demonstrate that most of the indices do not have a close relation to the actual service runtime. This means that the use of such indices as driving metrics for heuristics to optimize the performance can have serious drawbacks and platforms can be wrongly assessed to be overloaded or idle. This can reduce the overall performance of the services significantly, since requests can be sent in error to supposedly idle servers, which are actually overloaded. Alternatively, nodes can be considered overloaded where in fact they are not. In this case, unnecessary nodes may be used raising both the execution costs and the overall energy consumption.

The *JMX Average Time* index proved to be the best in terms of significance to estimate the service runtime, considering all the six scenarios of the experiments and the multiple linear regression model based on the stepwise-AIC method. Its significance changed from 46.0% up to 78.2% (64.7% on average) in those scenarios. The *JMX Average Time* index was also able to simulate the service runtime with high accuracy in relation to real runtime, obtaining an excellent coefficient of determination equivalent to 99.5%. *Swap Used* showed a good coefficient of determination equivalent to 90.3%, due to the scenario with the *overloaded* platform executing the memory-bound service. It also showed a null variation for other scenarios.

Other indices showed a smaller significance compared to *JMX Average Time* in the same scenarios. In some cases the significance was only smaller, as in the case of the *Swap Used* for all services with the *overloaded* platform. However, in other cases (*Amount of Requests* for the CPU-bound service, for instance), *Swap Used* was null. The simulations of the service runtime using some of these indices confirmed their relatively limited importance, with coefficients of determination indicating significance from 20% to 40%.

The *JMX Average Time* index is more stable than the other indices studied in most cases. It is stable for all scenarios that were considered, being in some cases even better than the stability of the runtime service. The stability of the other indices changed significantly depending on the scenario used. This stability behaviour for the other indices in different scenarios restricts the practical use of such indices because they are not able to correlate to runtime service, a more stable metric, in an appropriate way. The instability could be attenuated by using a larger window of samples in the EMA. However, all results in this paper used EMA and increasing the sample window size will also reduce the accuracy of the index, since it will introduce a longer delay before changes in the current workload are reflected in the index.

The main contribution of this paper is to demonstrate by means of experimental studies that the metrics commonly used by performance-estimating heuristics do not reflect, in practice, the actual performance. The results show that the mistaken use of such indices can lead to decisions that will have a negative impact on both service performance and execution cost.

Another contribution of this paper is the proposal of the *JMX Average Time* metric, a novel load index which is independent of the platform and to the demand generated by services. The *JMX Average Time* index is tightly-coupled to the service runtime, a widely used performance metric for computing platforms.

Future work will be directed to the following objectives: 1) applying the results of this paper to the ***Jerrymouse*** project [20] for the development of advanced request distributing policies on web service clusters; and 2) establishing a methodology to evaluate load indices in a comparable and standardized way.
